# Safety and efficacy of percutaneous electrocoagulation haemostasis in the treatment of grade IV haemorrhagic cystitis after allogeneic haematopoietic stem cell transplantation in children: a retrospective analysis

**DOI:** 10.1186/s13052-023-01470-3

**Published:** 2023-06-05

**Authors:** Hai-chao Liu, Yun-bo Yang, Peng Zhang, Jia-xing Zhang, Zhi-sheng Pei, Bo-wen Chen, Gui-qian Liu, Hui Li

**Affiliations:** 1 Department of Urology, Hebei Yanda Hospital, Langfang, 065201 China; 2 Department of Urology, Beijing Chaoyang Hospital of Capital Medical University, Beijing, 100020 China

**Keywords:** Children, Haematopoietic stem cell transplantation, Severe haemorrhagic cystitis, Endoscopic electrocoagulation

## Abstract

**Background:**

To investigate the efficacy and safety of endoscopic electrocoagulation haemostasis via a percutaneous transhepatic approach for the treatment of grade IV haemorrhagic cystitis (HC) after allogeneic haematopoietic stem cell transplantation (allo-HSCT) in children.

**Methods:**

The clinical data of 14 children with severe HC, who were admitted to Hebei Yanda Hospital between July 2017 and January 2020, were analysed retrospectively. There were nine males and five females, with an average age of 8.6 years (range: 3 to 13 years). After an average of 39.6 (7 to 96) days of conservative treatment in the hospital’s haematology department, the bladders of all patients were filled with blood clots. A small 2-cm incision was made in the suprapubic area to enter the bladder and quickly clear the blood clots, and a percutaneous transhepatic approach to electrocoagulation and haemostasis was performed.

**Results:**

In the 14 children, a total of 16 operations were performed, with an average operation time of 97.1 (31 to 150) min, an average blood clot of 128.1 (80 to 460) mL and an average intraoperative blood loss of 31.9 (20 to 50) mL. There were three cases of postoperative bladder spasm remission after conservative treatment. During the follow-up period of 1 to 31 months, one patient improved after one operation, 11 patients were cured after one operation, and two patients were cured after recurrent haemostasis by secondary electrocoagulation, four of whom died of postoperative non-surgical blood-related diseases and severe lung infections.

**Conclusion:**

Percutaneous electrocoagulation haemostasis can quickly remove blood clots in the bladders of children after allo-HSCT with grade IV HC. It is a safe and effective minimally invasive treatment.

## Background

In China, the incidence of leukaemia in children (under 15 years of age) is four to five in every one hundred thousand [1]. At present, the most effective treatment for leukaemia is allogeneic haematopoietic stem cell transplantation (allo-HSCT), in which haemorrhagic cystitis (HC) is a common complication [2, 3]. Haemorrhagic cystitis is clinically characterised mainly by the presence of lower urinary tract syndrome and includes urinary tract irritation symptoms, such as haematuria, frequent urination, urgency and dysuria [4]. In mild cases, it manifests as microscopic blood in the urine, while in severe cases, large blood clots may appear, causing urinary tract obstruction and even renal failure, seriously affecting the prognosis and health of patients.

Haemorrhagic cystitis is one of the most common complications after allo-HSCT, with an incidence of 3% to 35% [5, 6]. Severe HC leads to bladder blockage, which not only causes pain to the child but also seriously endangers their life, and surgical treatment is often required in clinical practice [7]. Currently, commonly used surgical treatments include cystoscopic electrocoagulation haemostasis and selective bladder artery or internal iliac artery embolisation [8, 9]. Cystoscopy enables the direct observation of intravesical lesions and the rapid removal of both intravesical blood clots and haemostasis in a short period, which can effectively treat severe HC. However, due to the small urethra in children and the lack of paediatric urological devices in some hospitals, minimally invasive surgery for severe HC in children has been limited [10]; furthermore, the blood clots blocking the bladders of such patients are more viscous and often cannot be removed in a timely and effective manner by transurethral surgery. According to the special physiological anatomy of children and experience in the treatment of bladder blockages in adults with severe HC [11], we chose to treat children with severe HC using a percutaneous route for clot removal in conjunction with electrocoagulation for haemostasis. No such route has been reported, so this study retrospectively analysed the clinical data of 14 children with severe HC and bladder blood clot occlusion after HSCT who were cured by percutaneous transurethral resectoscope electrocoagulation haemostasis in Hebei Yanda Hospital during the past three years. The report is as follows:

## Materials and methods

### Clinical data

The clinical data of 14 children (9 males and 5 females, aged 3 to 13 years, with a mean age of 8.6 years) who developed severe HC after allo-HSCT treatment in Hebei Yanda Hospital from July 2017 to January 2020 were reviewed. Inclusion criteria: haemorrhagic cystitis after allo-HSCT; conscious and stable vital signs; normal communication and cognitive ability; complete clinical data available for reference; voluntary participation in the study. Exclusion criteria: severe heart and lung failure; a history of bladder disease; a history (or family history) of mental illness.

There was one case of aplastic anaemia, four cases of acute myelogenous leukaemia and nine cases of acute lymphoblastic leukaemia. The mean time of onset was 96.3 (39 to 177) days after allo-HSCT. The mean time for HC to progress to severe HC after conservative treatment was 39.6 (7 to 96) days. All patients had been treated previously with continuous bladder irrigation with normal saline and repeated syringe aspiration and still had haematuria with difficult urination or ureteral obstruction and lower abdominal distension and discomfort. Physical examination showed a bulging bladder area, significant tenderness when touched and dullness under percussion. Ultrasound results showed a large blood clot in the bladder, with a volume of 80 to 460 mL. Preoperative WBC: (2 to 5) × 10^9^/L; haemoglobin: 51 to 105 g/L; platelets: (31 to 229) × 10^9^/L (see Table 1).

**Table 1:** Basic information of patients

Patient numbers	Gender	Age (years)	Bladder capacity (ml)	Operative duration (min)	Bleeding (ml)	Blood clot (ml)	Causes of transplantation	Transplant Time (min)	HB preoperative (g/L)	PLT preoperative (10^9^ /L)	Outcome	Postoperative flush time (days)	Time to progression from HC with conservative treatment to severe HC (days)	Efficacy	Efficacy (reoperation)
1	Male	9	300	120	50	200	Acute lymphoblastic leukemia	177	105	114	Died	35	51	CR	
2	Female	13	420	123	50	300	Acute lymphoblastic leukemia	70	86	78	Cured	3	31	CR	
3	Male	8	270	150	20	200	Acute myeloid leukaemia	69	67	229	Cured	20	21	PR	CR
4	Male	3	120	125	20	80	Acute lymphoblastic leukemia	76	65	117	Cured	15	50	CR	
5	Female	12	390	113	10	300	Acute lymphoblastic leukemia	120	88	121	Cured	25	90	CR	
6	Male	13	420	105	50	200	Acute lymphoblastic leukemia	120	69	67	Died	34	60	PR	CR
7	Male	5	180	56	50	100	Acute myeloid leukaemia	150	58	36	Recovered	27	96	CR	
8	Female	12	390	31	20	200	Aplastic anemia	150	80	79	Cured	30	30	CR	
9	Male	5	180	110	30	160	Acute lymphoblastic leukemia	60	93	131	Cured	7	15	CR	
10	Female	8	270	100	30	200	Acute lymphoblastic leukemia	65	81	116	Cured	11	40	CR	
11	Male	13	420	75	20	400	Acute myeloid leukaemia	39	86	31	Death	30	7	CR	
12	Female	4	150	85	20	150	Acute lymphoblastic leukemia	98	112	143	Cured	98	37	CR	
13	Male	8	270	115	30	200	Acute myeloid leukaemia	114	77	20	Cured	14	7	CR	
14	Male	7	240	80	30	210	Acute lymphoblastic leukemia	40	97	72	Died	7	20	CR	

### Diagnosis of HC

Haemorrhagic cystitis is persistent haematuria and lower urinary tract symptoms that occur when other disease conditions are excluded (e.g. vaginal bleeding, systemic bleeding body, urinary tract infection) [12]. The criteria for grading the diagnosis of HC (Droller criteria) are as follows: grade I: microscopic haematuria; grade II: macroscopic haematuria; grade III: macroscopic haematuria with small blood clots; grade IV: macroscopic haematuria with blood clots blocking the urethra and requiring the use of an instrument for blood clot clearance [13]. In this study, a grade IV blood clot blockage of the bladder was classed as severe HC.

### Surgical methods

Each child was placed in the supine position, and, after general anaesthesia, a longitudinal incision of about 2 cm was made in the suprapubic midline. Each layer was incised successively, and the peritoneal reflection was pushed open. Allis forceps were used to lift the anterior bladder wall, and an electrotome was used to create an incision. Oval curved forceps were used to remove the blood clot blockage rapidly. A 12-mm laparoscopic trocar was indwelled through the bladder incision, and a suture was fixed. An F26.5 plasmakinetic resectoscope was inserted via the trocar to fill the bladder bleeding under continuous irrigation, the electrocoagulation points were punctated accurately, and normal bladder mucosa was preserved as much as possible. Drainage was performed from the lateral aperture of the trocar to create circulation and keep the surgical field clear. After observing that there was no obvious bleeding point in the bladder mucosa, an F20 three-lumen urinary catheter was selected for the cystostomy tube, continuous bladder irrigation was performed, and the incision was sutured layer by layer.

### Observation indicators

The main observation indicator of this study was clinical efficacy, and the secondary observation indicators included the intraoperative indexes (operation time, intraoperative bleeding volume, clot removal volume) of patients and the occurrence of postoperative complications.

The criteria for judging efficacy were as follows:

Complete remission (CR): the disappearance of haematuria and no recurrence at long-term follow-up. Partial remission (PR): a significant reduction of haematuria without transfusion therapy, or asymptomatic persistent microscopic haematuria. Non-remission (NR): no reduction of haematuria in the child or reappearance one week after the disappearance of postoperative sarcoid haematuria. The cure rate was CR / (CR + PR + NR), and the efficiency rate was (CR + PR) / (CR + PR + NR).

### Statistical analysis

All data were statistically analysed using SPSS16 software. Enumeration data are presented as percentages, and measurement data are presented as means ± standard deviation.

## Results

A total of 16 operations were performed in 14 patients, with an average operation time of 97.1 (31 to 150) min and an average blood loss of 31.9 (20 to 50) mL; the average removed blood clots were 128.1 (80 to 460) mL. Intraoperatively, random bladder mucosal biopsies were performed on two patients who were positive for BK virus DNA. Postoperative continuous bladder irrigation was performed routinely, and the average bladder irrigation time was 25.1 (3 to 98) days. During the follow-up period of 1 to 31 months, three children had postoperative bladder spasms, which were relieved after the oral administration of M receptor blockers and the intravenous application of phloroglucinol; two patients had rebleeding and blood clot blockages after one month and were cured after reoperation, of which one had rejection after bone marrow transplantation and one had leukaemia recurrence. One child still had haematuria one month after surgery, but the symptoms of haematuria were relieved compared with those before surgery, and continuous bladder irrigation was performed. The remaining 13 children underwent slow irrigation via the bladder. After no significant bleeding, the irrigation was stopped, and the stoma tube was clamped. After urinating spontaneously for two weeks without bleeding, the fistula was clamped shut. Patients urinated on their own for two weeks without bleeding; the F8 urinary catheter was left in place, the cystostomy tube was removed, and the fistula was plugged with oil and sand strips after it healed. During the follow-up period, four patients died of non-surgery-related underlying haematological diseases and pulmonary infection after surgery, and none of the patients had complications, such as bladder contracture. The primary surgery cure rate was 78.6%, the secondary surgery cure rate was 92.9%, and the total effective rate was 100.0%.

## Discussion

Haemorrhagic cystitis is a common complication after HSCT, in which the patient’s age at the time of allo-HSCT, the type of transplantation, GVHD and viral infection have some correlation with HC development [14]. Severe HC leads to bladder clot blockage when the condition is not relieved after conservative treatment, and clot retention places the patient at risk of persistent bleeding and urinary tract infection. In extreme cases, it leads to bladder overdistension and rupture, severely affecting the patient’s renal function and even becoming life-threatening [15].

During treatment, we found that the bladder mucosa of patients with severe HC showed diffuse lesions, the time of electrocoagulation haemostasis was relatively long, and prolonged transurethral approach electrocoagulation haemostasis could damage the urethral mucosa. The application of an electrotome under direct vision for bladder mucosal haemostasis has the problems of large trauma, unclear vision and incomplete haemostasis. Therefore, we used percutaneous transurethral resectoscope electrocoagulation for haemostasis in the treatment of severe HC in children. In the follow-up period of this study, one patient improved after one operation, 11 patients were cured after one operation, and two patients with recurrence were cured after secondary electrocoagulation haemostasis, with an overall response rate of 100.0%. In all patients, the cystostomy tube was removed successfully after surgery, and the stoma healed well. However, Kaplan et al. [16] retrospectively analysed 12 patients with HC after HSCT and reported a cystoscopic blood clot clearance rate of 61%. In this study, the intravesical blood clots existed for a long time and even reached 460 mL. The blood clots were removed rapidly by this method, and it was observed that they had been partially organised after removal, although the blood clots could not be removed by aspiration with a simple blood-drawing device. This method involves no transurethral surgery and avoids damage to the urethral mucosa, postoperative urination pain, urethral stricture and other complications. A resectoscope can flexibly enter and exit the trocar indwelled during surgery, and damage to the bladder wall can be reduced. Drainage can be performed through the side holes of the trocar to create circulation, and the bladder can be filled under continuous irrigation, fully exposing the bladder mucosa and bilateral ureteral orifices, precisely punctuating electrocoagulation bleeding spots and preserving the normal bladder mucosa as much as possible. At the same time, it is important to avoid overfilling the bladder and causing both new bleeding and the extravasation of irrigation fluid.

After the operation, we found that small blood clots were often discharged from the cystostomy tube, and there was a risk of urethral blockage due to the small size of the indwelling catheter via the urethral route for haemostasis treatment. The children often complained of urethral pain and other discomforts from the indwelling catheter via the urethra; therefore, in this study, the F20 three-lumen urethral tube was chosen as the cystostomy tube, with continuous bladder irrigation used to prevent small blood clots blocking the urethra and causing rebleeding. Postoperative re-examination showed that no patients had incision infection, so this method was safe and effective.

Recurrence was possibly related to postoperative catheter-related infection in two of the re-operated children. The urinary catheters of these two children were obtained for bacterial culture during the second operation, of which *Klebsiella pneumoniae* was cultured in one case, and *Staphylococcus hominis* subsp. *hominis* was cultured in another case. The causes of infection were analysed as follows: (i) patients after HSCT are mostly immunosuppressed and prone to infection [17]; (ii) catheter material: Latex catheters were used in both patients after surgery; the literature reports that catheter-related infection occurs more with latex catheters than with silicone catheters under the same use time [18]. Therefore, to reduce the recurrence of HC caused by postoperative catheter-related infection, it is recommended that ① preoperative and postoperative urine cultures are performed on children, children with indwelling catheters should have their catheters taken for bacterial culture at the same time, and early treatment with sensitive antibiotics should be given according to the culture results, and ② postoperative silicone catheters should be left in place.

## Conclusion

In summary, the percutaneous approach of electrocoagulation haemostasis in the treatment of severe HC after HSCT in children can quickly remove blood clot blockages in the bladder and solve the problem of bladder haemostasis without causing bladder contracture after surgery. It is a safe and effective minimally invasive method. However, the number of cases in this study was small, and more clinical data are needed for further verification of the results.

## Data Availability

All data generated or analyzed during this study are included in this published article.
